# Nurses and Physicians Attitudes toward Nurse-Physician Collaboration: A Survey from Gaza Strip, Palestine

**DOI:** 10.1155/2017/7406278

**Published:** 2017-02-23

**Authors:** Aymen Elsous, Mahmoud Radwan, Samah Mohsen

**Affiliations:** ^1^Quality Improvement and Infection Control Office, Shifa Medical Complex, Al Rimal, Gaza City, Gaza Strip, State of Palestine; ^2^Department of Health Management and Economics, School of Public Health, Tehran University of Medical Sciences, International Campus, Tehran, Iran; ^3^General Directorate of International Cooperation, Ministry of Health, Al Rimal, Gaza City, Gaza Strip, State of Palestine

## Abstract

Interprofessional collaboration and teamwork between nurses and physicians is essential for improving patient outcomes and quality of health services. This study examined the attitudes of nurses and physicians toward nurse-physician collaboration. A cross-sectional study was conducted among nurses and physicians (*n* = 414) in two main referral public hospitals in the Gaza Strip using the Arabic Jefferson Scale of Attitude toward Physician-Nurse Collaboration. Descriptive statistics and difference of means, proportions, and correlations were examined using Student's *t*-test, one-way ANOVA, and Pearson correlation and *p* < 0.05 was considered as statistical significant. Response rate was 42.8% (75.6% for nurses and 24.4% for physicians). Nurses expressed more positives attitudes toward collaboration than physicians (M ± SD on four-point scale: 3.40 ± 0.30 and 3.01 ± 0.35, resp.) and experience duration was not proved to have an interesting influence. Teamwork approach in the professional practice should be recognized taking into consideration that the relationship between physicians and nurses is complementary and nurses are partners in patient care.

## 1. Introduction

The interprofessional collaboration between physicians and nurses is crucial and has been highlighted in different contexts [1, 2]. Collaboration, between physicians and nurses, means cooperation in work, sharing responsibilities for solving problems, and making decisions to formulate and carry out plans for patient care [3]. Although the provision of healthcare is becoming more complex, collaboration among healthcare workers can be a path to improve the quality of healthcare services especially in hospitals in which environment is characterized by ongoing interaction among professionals. Nurse-physician collaboration and teamwork can improve patient outcomes and lower healthcare cost [4], increase job satisfaction [5], and maintain patients' safety [6]. The communication between nurses and physicians is considered a principal part of the information flow in healthcare; meanwhile the growing evidences show that improper or poor communication can create a chronic state of conflict between nurses and physician leading to increase in the medical errors and poor outcomes [4, 7]. Furthermore, it has been shown that unsatisfactory interprofessional relationships between physicians and nurses partially contributed to shortage of nurses and enforced nurses to leave their professions [8].

Nurses and physicians extremely contribute to the patient care but often do not appreciate the role of each other [9]. In previous studies, doctors and nurses viewed collaboration differently; doctors view collaboration as following the instructions and the orders, while nurses view it as a complementary role more significantly than physicians do [10]. Bujak and Bartholomew suggest that presently “the two most important people responsible for patient care are the nurses and the physicians, but they often do not talk to each other properly, and when they do, the interchange is often dysfunctional” [11]. Traditionally, relationship between the physicians and the nurses is hierarchical and is characterized by doctors' dominance and nurses are viewed as assistant rather than a partner of holistic patient care [12].

In the Palestinian health context, the physicians' approach is medically dominant comparing to nurses. Physicians have historically been in a position of greater authoritative power. The organizational structure endorsed by the Ministry of Health has left the nursing profession under the description as nonindependent body where nurses are organizationally managed by physicians and technically have to implement their orders. Such state can make the nurses feel less autonomy and respect and could create less cooperative relationship. To the best of our knowledge, this is the first study assessing nurses-physician collaboration using the Arabic version of the Jefferson Scale of Attitude toward Nurse-Physician Collaboration. Based on the available literature, sociodemographic variables such as gender and age are not a significant influencer like experience at work and cultural factors in determining the and shaping attitudes toward nurse-physician collaboration [6, 13–15]. The purpose of this study was to describe the attitudes of nurses and physicians toward nurse-physician collaboration. In addition, the following three hypotheses examining underlying factors affecting nurse-physician collaborative relationships were also examined:

(H1) The nurses have more positive attitudes toward nurse-physician collaboration than the physicians.
(H2) Female physicians have more positive attitudes toward nurse-physician collaboration than male physicians.
(H3) Attitude toward nurse-physician collaboration is correlated with length of experience at work.

These hypotheses are based on role theory [16, 17] which indicates that everyone activity is according to socially defined categories and interaction between persons and is directed by the position each person holds in a social or professional relationship, and therefore interactions do not occur if persons fail to assume their role. In this theory there are behaviors, expectations, and competence required for each role. To describe collaboration as a behavior, role theory could be used to describe the complexities of roles of the collaborating providers.

From the standpoint of role theory, nurses are more likely to have more positive attitude than physicians toward nurse-physician collaboration consistent with previous findings [14, 18]. This hypothesis is derived from the historical role differentiation which assigns lower professional status to nurses and cultural norms that nurses are physicians' followers or order implementers.

The second hypothesis examines the differences between female and male physicians toward physician-nurse collaboration. This hypothesis is based on the gender influence role in the male-foremost profession of medicine and the female-foremost profession of nursing. The third hypothesis explores correlation between attitudes toward collaboration and work experience. The basis of this hypothesis is that nurses and physicians become familiar with each other and feel that they are part of a large family when working together for a long time. The bickering resulted from perception of inferiority of nursing in the beginning of new employment melts with time.

## 2. Materials and Methods

### 2.1. Design, Setting, and Period of Study

This was a cross-sectional design conducted in a two main referral hospitals in the Gaza Strip and impatient wards were classified as surgical, medical, and maternity. The two hospitals are the largest and biggest and serve half of Gaza Strip inhabitants (*n* = ~one million). The data collection continued for four months from November 2015 to February 2016.

### 2.2. Sample and Sampling

All eligible nurses and physicians were invited to participate in the study. They should be formal employees with at least 6-month experience in their work place. The total number of nurses and physicians available at the time of study was 543 and 423, respectively. All eligible nurses and physicians were recruited for the study (census sample).

### 2.3. Measures

We used the Arabic version of the Jefferson Scale of Attitude toward Physician-Nurse Collaboration. We translated the JSAPNC into Arabic following the recommended guidelines [19] and tested its validity and reliability which was shown to be a psychometric soundness tool. The Cronbach alpha was 73.2 for the entire model (74.7–89.5) and test-retest reliability was 70.9 and 69.7, respectively. The item content validity index and scale content validity index ranged from 0.77 to 1.00 and 0.88 to 0.94, respectively.

The questionnaire has two parts: Demographic characteristics included age, gender, experience at work, educational level, and place of work. The second part is 15 questions constituting 4 factors slightly modified from the original scale: (1) nurse-physician collaboration (items 3, 4, 5, 7, 9, 11, 12, and 13), (2) doctor's authority (items 14 and 15), (3) shared education (items 1, 2, and 6), and (4) nursing role in patient care (items 8 and 10).

The responses are measured on 4-point Likert scale: (1) = strongly disagree; (2) = disagree; (3) = agree; (4) = strongly agree. The two statements of factor (2) are reversed-scored, with a higher factor score given to a lower numerical answer and vice versa.

### 2.4. Ethical Consideration

A permission to conduct the study was obtained from the hospital administration after giving a short presentation on the objectives and expected results. Study's aim was explained to all participants' prior beginning of the study. Participation was voluntary based and their identities were anonymous. To ensure maximum confidence of data, the questionnaires were placed in researchers' closet.

### 2.5. Statistical Analysis

The statistical package for social sciences (SPSS) version 22 was used for analysis. Data were presented in form of frequencies, means, standard deviation, and percentage for quantitative variables. *t*-test was used to make comparison between two groups and one-way ANOVA among three groups and *p* < 0.05 was considered statistically significant. Person correlation analysis was used for the assessment of the interrelationships among quantitative variables.

### 2.6. Operational Definition

Positive attitude toward nurse-physician collaboration: the higher the total scores on this scale, the more positive the respondent's attitude toward physician-nurse collaboration

## 3. Results

### 3.1. Demographic Characteristics

Out of 966 recruited nurses and physicians 414 responded (42.85%) of which 101 (24.4%) were physicians and 313 (75.6%) were nurses. The response rate among nurses and physicians was 57.64% and 23.87%, respectively. Two hundred and seventy-nine (67.4%) were males, and 217 were below 35 years old (52.4%). Majority of nurses had less than 10 years' experience while half of the physicians have experience between 11 and 20 years (Table 1).

### 3.2. Differences in the Mean Values

The *t*-test analysis revealed significant differences in the attitude toward collaboration between physicians and nurses (*t*-test: 10.391; *p* < 0.001). The mean total score, on the four-point scale, for nurses was 3.40 (SD: 0.30) compared to 3.01 (SD: 0.35) for physicians (Table 2). Nurses scored higher than physicians in the four subscales of the questionnaire which was statistically significant (*p* < 0.001), indicating that the nurse's attitudes toward nurse-physician collaboration were more positive than the physician's. For instance, in the factor (2) “doctor's authority” (a higher factor score indicates a rejection of a totally dominant role by the physician in aspects of patient care), the nurses' mean score of 3.35 (SD = 1.38) was higher than the physicians (2.25, SD = 1.51) on the four-point scale.

The individual item scores and the item total correlation are also examined and are shown in Table 3. For each item, nurses scored higher and showed predisposition for collaboration better than physicians. The item total correlations supported the interitem relationship which is ranged between 0.26 and 0.68. The fourth question “there are many overlapping areas of responsibility between physicians and nurses” had the weakest correlation.

Tables 4 and 5 show the variations of the mean scores of nurses and physicians separately with regard to hospital departments. Generally, nurses in internal medicine wards had positive attitudes toward collaboration compared to nurses in the counterparts in surgical and maternity wards. They scored higher in all JSAPNC subscales but not with factor four “nursing role in patient care” which was only significant with factor two “doctors authority.” Post hoc Bonferroni test was performed to identify significance of the differences between the departments which revealed statistical significance between medical and maternity only (*p* < 0.05). As regards physicians, internal medicine physicians scored higher than their counterpart in surgical wards in all subscales but were not statistically significant at all (*p* > 0.05).

Table 6 shows the positive correlation between JSAPNC and the age (0.127). The two factors which showed positive correlations were doctor's authority (0.142) and shared education (0.169). In return, experience was found to have positive correlation only with shared education factor (0.108).

## 4. Discussion

This research adds up to date knowledge on nurse-physician collaboration in the inpatient settings in the Gaza Strip. Collaboration requires mutual respects, open communication, and shared decision making power [20]. In collaboration, problem solving is a mutual effort in which no superiority is present in the relationship between physicians and nurses. Traditionally, physicians view nurses as subordinate in which they receive orders for implementation. This tradition views on the physician-nurse relationship can affect health workers' attitudes toward collaboration.

We found that attitude toward collaboration between physicians and nurses is significantly different and nurses showed more favorable attitudes than physicians, which is in line with the idea presented by Hojat et al. [14] “principle of least interest.” The findings are further supported by previous similar researches conducted in a hospital based setting [1, 21, 22]. Other studies showed different results in which physicians in ICU units had more positive attitudes toward collaboration than nurses [23, 24]. The literature attribute the nurse-physician relationship to hierarchical model of patient care which means nurses are doctors' assistant and are viewed as subordinate. Studies of Hojat et al. [14, 18] showed that American nurses expressed more positive attitudes toward collaboration than their counterparts in Italy and Mexico. This is because the American nurses followed complementary model of professional roles rather than hierarchical model of practice in the counterpart. However, physicians can easily acknowledge the nurses' role. MacDonald and Katz [25] and Barrere and Ellis [26] stated that when knowledge concerning nursing role increases, significant favorable changes in the nurses' attitude toward collaboration can happen. Therefore, limited knowledge of nursing roles negatively affects the physicians' willingness to practice collaboration.

Analysis of the Jefferson subscales revealed that the nurses scored higher than physicians in all subscales including the psychosocial aspect of care. This means that nurses have more favorable attitudes toward contribution in the psychosocial aspect of care. Furthermore, they showed a big rejection of dominant physician's role. The findings are in line with previous studies from Egypt, Sweden, and America [6, 18, 27].

In the present study, more positive attitudes and orientations toward “shared education” are explicitly presented in item 2 from both nurses and physicians. The finding is consistent with previous publications by Thomson [21] and Sterchi [22].

Disagreement is obvious about nursing role in the patient care (items 8 and 10). Physicians scored above disagree while nurses scored above agree. This is similar to Sterchi study [22] who attributed it to the lack of organizational support for nurses' contribution in holistic model of the patient care.

This study revealed no differences in physicians' attitudes toward collaboration based on hospital departments. Similar findings were found with nurses except with factor (2) “doctor's authority” which was shown to be significant. Physicians and nurses of medical wards scored higher than their counterparts which indicated a more positive attitude toward nurse-physician collaboration. This could be attributed to the nature of medical wards setting in Gaza Strip which comprise various specialized disciplines including but not limited to endocrinology, rheumatology, gastroenterology, pulmonology, and neurology. This means that physicians and nurses have to work and coordinate their care with other care providers which may enhance teamwork and collaboration. A study by Chaboyer and Patterson [28] found nurses who work in specialized wards perceived better attitudes toward physician-nurse collaboration than general nurses.

As regards the correlation between JSAPNC subscales with age and years of experience at work, this study revealed that a more positive attitude toward collaboration is correlated with the age of the nurses and physicians in contrast to Ward et al's study [2] and El Sayed and Sleem findings [6]. A possible explanation could be that mentality, thinking, and acceptability of others' role within the complementary model of care practice are matured when age increases. No correlation was found between length of experience and attitudes toward collaboration which is against the findings of Sterchi's [22] and El Sayed's and Sleem's study [6].

This study has limitations: (1) The physician-nurse attitudes toward collaboration are complex in nature and are determined by many factors. The JSAPNC relied on caregivers' perceptions and may not reflect the actual complexity of this relationship. Therefore, interpretation of results should be considered with cautions. (2) The study was based in two public hospitals only although they are the largest and the biggest, but making the generalization statement sensitive. (3) We do not know whether the nonresponders' perceptions are consistent with surveyed nurses and physicians.

## 5. Conclusion

The study's findings supported one of the three research hypotheses. The first confirmed hypothesis: nurses have more positive attitudes toward collaboration than physicians. We were unable to confirm the second hypothesis because the sample of female physicians is not enough and cannot be examined. The third unconfirmed hypothesis is that work experience has strong correlation with attitudes toward collaboration.

## Figures and Tables

**Table 1:** Sociodemographic characteristics of participants (*N* = 414).

Variables	Physicians (*N* = 101) (%)	Nurses (*N* = 313) (%)	Total (*N* = 414) (%)
Gender			
Male	97 (96)	182 (58.1)	279 (67.4)
Female	4 (4)	131 (41.9)	135 (32.6)
Age			
≤35 years	20 (20.2)	197 (63.4)	217 (52.4)
>35 years	79 (79.8)	114 (36.6)	193 (46.6)
Place of work			
Surgical	77 (76.2)	147 (47.0)	224 (54.1)
Internal medicine	24 (23.8)	81 (25.9)	105 (25.4)
Maternity	0	81 (25.9)	81 (19.6)
Years of experience			
≤10 years	33 (32.6)	200 (63.9)	233 (56.2)
11–20 years	57 (56.4)	63 (20.1)	120 (29)
>21 years	11 (10.9)	50 (16.0)	61 (14.4)
Education			
Diploma	—	116 (37.1)	117 (28.3)
Bachelor	28 (27.6)	164 (52.4)	192 (46.3)
Master	41 (40.6)	21 (6.7)	62 (15.0)
Ph.D.	7 (6.9)	4 (1.3)	11 (2.7)
Board	25 (24.8)	—	25 (6)

**Table 2:** Mean values and differences between physicians and nurses according to JSAPNC domains.

Factors	Professions	M (SD)	SEM	*t*	df	*p* value
F1: physician-nurse collaboration	Physicians Nurses	26.79 (3.14) 28.00 (2.74)	0.319 0.159	3.641	394	<0.001
F2: doctor's authority	Physicians Nurses	4.51 (1.51) 6.67 (1.38)	0.150 0.078	13.294	412	<0.001
F3: shared education	Physicians Nurses	8.63 (1.78) 9.97 (1.34)	0.179 0.076	6.868	135	<0.001
F4: nursing role in patient care	Physicians Nurses	5.36 (1.38) 6.34 (1.29)	0.138 0.073	6.489	408	<0.001
Overall attitude	Physicians Nurses	45.28 (5.30) 51.12 (4.55)	0.547 0.265	10.391	387	<0.001

**Table 3:** JSAPNC individual item mean scores.

JSAPNC questions		Physicians	Nurses	Totals	Corrected item, total correlation
		M (SD)	M (SD)	M (SD)	
3	A nurse should be viewed as a collaborator and colleague with a physician rather than his/her assistant	3.55 (0.64)	3.74 (0.48)	3.69 (0.53)	0.45
5	Physicians should be educated to establish collaborative relationships with nurses ^*∗*^(building collaborative relationship should be concerned by physician)	3.58 (0.53)	3.59 (0.52)	3.59 (0.52)	0.35
4	There are many overlapping areas of responsibility between physicians and nurses	3.01 (0.78)	3.15 (0.69)	3.11 (0.71)	0.26
7	Nurses should also have responsibility for monitoring the effects of medical treatment	3.09 (0.89)	3.33 (0.63)	3.27 (0.71)	0.42
12	Nurses should be involved in making policy decisions concerning the hospital support services upon which their work depends	3.27 (0.66)	3.54 (0.54)	3.48 (0.58)	0.54
11	Nurses should clarify a physician's order when they feel that it might have the potential for detrimental effects on the patient	3.42 (0.63)	3.54 (0.55)	3.51 (0.57)	0.41
9	Nurses should be involved in making policy decisions affecting their working conditions	3.29 (0.52)	3.58 (0.56)	3.51 (0.56)	0.48
13	Nurses should be accountable to patients for the nursing care they provide	3.54 (0.59)	3.47 (0.53)	3.49 (0.55)	0.35
14	The primary function of the nurse is to carry out the physician's orders	2.43 (0.87)	3.27 (0.79)	3.07 (0.88)	0.62
15	Doctors should be the dominant authority in all health care matters	2.07 (0.93)	3.39 (0.76)	3.07 (0.98)	0.68
6	Physicians and nurses should contribute to decisions regarding the hospital discharge of patients	2.31 (0.81)	3.21 (0.71)	2.99 (0.83)	0.61
2	Interprofessional relationships between physicians and nurses should be included in their educational programs	3.37 (0.61)	3.43 (0.52)	3.41 (0.54)	0.37
1	During their education, medical and nursing students should be involved in teamwork in order to understand their respective roles	2.96 (0.9)	3.33 (0.64)	3.24 (0.73)	0.43
10	Nurses have special expertise in patient education and psychological counseling ^*∗*^(nurses have special expertise in patient education and psychological support)	2.74 (0.77)	3.26 (0.69)	3.14 (0.74)	0.58
8	Nurses are qualified to assess and respond to psychological aspects of patients' needs	2.62 (0.88)	3.07 (0.74)	2.96 (0.80)	0.57

^*∗*^Paraphrased sentence and adapted to Palestinian culture.

**Table 4:** Attitude of nurses toward nurse physician collaboration factors according to place of work.

JSAPNC factors	Hospital departments			*p* value
	Surgical (*n* = 147) M (SD)	Medical (*n* = 81) M (SD)	Maternity (*n* = 81) M (SD)	
F1: physician-nurse collaboration	27.82 (2.64)	28.37 (2.53)	27.86 (3.05)	0.322
F2: doctor's authority	6.58 (1.44)	7.07 (1.23)	6.43 (1.37)^*∗*^	0.014
F3: shared education	9.91 (1.38)	10.12 (1.41)	9.93 (1.22)	0.507
F4: nursing role in patient care	6.34 (1.12)	6.14 (1.58)	6.55 (1.23)	0.136
Total	50.75 (4.37)	51.67 (4.65)	51.10 (4.77)	0.362

^*∗*^Significant between medical and maternity (*p* < 0.05) (post hoc, Bonferroni test).

**Table 5:** Attitude of physicians toward nurse physician collaboration factors according to place of work.

JSAPNC factors	Hospital departments		df	*t*	*p* value
	Surgical	Medical			
	M (SD)	M (SD)			
F1: physician-nurse collaboration	26.70 (3.32)	27.08 (2.55)	95	0.509	0.612
F2: doctor's authority	4.49 (1.42)	4.58 (1.79)	99	0.253	0.801
F3: shared education	8.45 (1.80)	9.20 (1.64)	97	1.823	0.071
F4: nursing role in patient care	5.35 (1.43)	5.37 (1.27)	98	0.060	0.952
Total	45.04 (5.33)	46.04 (5.26)	92	0.758	0.435

**Table 6:** Correlation coefficient between nurse physician collaboration factors and demographic characteristics.

JSAPNC factors	Demographic characteristics	
	Age (*r*) (*p* value)	Experience (*r*) (*p* value)
F1: physician-nurse collaboration	0.045 (0.37)	0.012 (0.808)
F2: doctor's authority	0.142^*∗∗*^ (0.004)	0.023 (0.640)
F3: shared education	0.169^*∗∗*^ (0.001)	0.108^*∗*^ (0.029)
F4: nursing role in patient care	−0.063 (0.203)	−0.010 (0.843)
Total	0.127^*∗*^ (0.012)	0.034 (0.508)

^*∗∗*^Correlation is significant at the 0.01 level (2-tailed).

^*∗*^Correlation is significant at the 0.05 level (2-tailed).
